# A soybean MADS-box protein modulates floral organ numbers, petal identity and sterility

**DOI:** 10.1186/1471-2229-14-89

**Published:** 2014-04-02

**Authors:** Fang Huang, Guangli Xu, Yingjun Chi, Haicui Liu, Qian Xue, Tuanjie Zhao, Junyi Gai, Deyue Yu

**Affiliations:** 1 National key laboratory of crop genetics and germplasm enhancement, National Center for Soybean Improvement, Nanjing Agricultural University, Nanjing 210095, China

**Keywords:** Fertility, Floral organ number, Petal identity, *Glycine max*, MADS-box transcription factors

## Abstract

**Background:**

The MADS-box transcription factors play fundamental roles in reproductive developmental control. Although the roles of many plant MADS-box proteins have been extensively studied, there are almost no functional studies of them in soybean, an important protein and oil crop in the world. In addition, the MADS-box protein orthologs may have species-specific functions. Controlling male fertility is an important goal in plant hybrid breeding but is difficult in some crops like soybean. The morphological structure of soybean flowers prevents the cross-pollination. Understanding the molecular mechanisms for floral development will aid in engineering new sterile materials that could be applied in hybrid breeding programs in soybean.

**Result:**

Through microarray analysis, a flower-enriched gene in soybean was selected and designated as *GmMADS28*. GmMADS28 belongs to AGL9/SEP subfamily of MADS-box proteins, localized in nucleus and showed specific expression patterns in floral meristems as well as stamen and petal primordia. Expression of *GmMADS28* in the stamens and petals of a soybean mutant NJS-10Hfs whose stamens are converted into petals was higher than in those of wild-type plants. Constitutive expression of *GmMADS28* in tobacco promoted early flowering and converted stamens and sepals to petals. Interestingly, transgenic plants increased the numbers of sepal, petal and stamen from five to six and exhibited male sterility due to the shortened and curly filaments and the failure of pollen release from the anthers. The ectopic expression of *GmMADS28* was found to be sufficient to activate expression of tobacco homologs of SOC1, LEAFY, AGL8/FUL, and DEF. In addition, we observed the interactions of GmMADS28 with soybean homologs of SOC1, AP1, and AGL8/FUL proteins.

**Conclusion:**

In this study, we observed the roles of GmMADS28 in the regulation of floral organ number and petal identity. Compared to other plant AGL9/SEP proteins, GmMADS28 specifically regulates floral organ number, filament length and pollen release. The sterility caused by the ectopic expression of *GmMADS28* offers a promising way to genetically produce new sterile material that could potentially be applied in the hybrid breeding of crops like soybean.

## Background

Many transcription factors (TFs) that control floral development in model plants, such as Arabidopsis, have been identified and constitute a genetic “ABC model” for specifying four-whorl floral organs: sepals, petals, stamens, and carpels [1,2]. Most TFs in the “ABC model” are so-called MADS-box transcription factors, which play fundamental roles in floral developmental control [3-5]. The ABC model proposes three classes of genes, termed A, B, and C. A genes alone specify sepals, A and B genes together specify petals, and B gene expression in combination with a C gene specifies stamens. Carpel formation is dependent on C gene expression alone. In addition, the ABCD model was suggested when *FLORAL BINDING PROTEIN 11*(*FBP11*), termed a D-class gene, was confirmed to determine the ovule [6]. Although ABC genes are critical for floral organ identity, their expression levels are not sufficient to convert leaves into floral organs, indicating that other genes are required. Further studies in Arabidopsis have identified E genes (*SEPALLATA1–4*, previously named *AGL2*, *AGL4*, *AGL9*, and *AGL3*), which are required to specify the identity of all four whorls of the floral organs and floral meristem determinacy [7-11]. Accordingly, the ABCDE model was proposed [12-14].

The *SEPALLATA* (*SEP*) or *SEP*-like genes have been described necessary, albeit redundantly, for the normal development of petals, stamens, carpels, and sepals [8,13,15]. It has been proposed that the SEP proteins could constitute higher-order complexes with A, B, or C proteins [8,13,15], and at least one SEP protein is present in more than half of the 106 multimeric complexes identified through a large-scale yeast three-hybrid screen [16]. SEP proteins are necessary for the formation of the transcription factor complexes that control ovule development [17]. A single *sep* mutant shows either a subtle or no phenotype, whereas the *sep1/2/3/4* mutant displays indeterminate flowers composed of only leaf-like organs; even sepal development is dependent on SEP function [10]. Although *SEP4* is involved redundantly with the other *SEP* genes in the development of all floral organs, the *sep1/2/3/4* quadruple mutant exhibits the more extreme phenotype of the loss of floral meristem identity compared to the *sep1/2/3* triple mutants [10]. Additionally, the *SEP3* mutation alone leads to a partial transformation of the petals into sepals, suggesting that SEP3 plays more predominant role in floral organ development than other SEP proteins [10]. SEP3 strongly activates the expression of the A gene *AP1*, B gene *AP3*, and C gene *AG*; these factors are the major protein interaction partners of SEP3, suggesting that SEP3 can activate the flower developmental program by enhancing the expression of its interaction partners [18]. The mutation of a rice *SEP*-like gene, *OsMADS34*/*PAP2*, altered inflorescence morphology and increased the number of primary branches but decreased the number of secondary branches [19,20]. Moreover, *osmads34* mutant displayed a decreased spikelet number and had lemma/leaf-like elongated sterile lemmas. Similar to the *sep1/2/3/4* mutants of Arabidopsis, knock-down of the four rice *SEP*-like genes led to homeotic transformation of all the floral organs, except the lemma, into leaf-like organs, suggesting the conservation of *SEP*-like genes in specifying floral determinacy and organ identity in both eudicots and monocots [21].

The phenotypes of transgenic plants constitutively expressing *SEP* genes have been analyzed to further study their functions. Pelaz et al. [22] found that the overproduction of *SEP3* in Arabidopsis led to early flowering and that the secondary shoots were transformed into solitary flowers. Furthermore, the ectopic expression of *SEP3* was found to be sufficient to activate other B and C genes, and the constitutive expression of *SEP3* and *LEAFY*, a floral meristem identity gene, produced ectopic petals, carpels, and ovules outside of the floral context [23]. Zhao et al. [24] found that the ectopic expression of a wheat *SEP*-like gene, *TaMADS1*, caused early flowering and altered the development of all floral organs, including sepals converted into leaf-like structures and decreases in the petal and stamen number. The overexpression of *SEP3* genes from tobacco and rice in Arabidopsis or tobacco promotes early flowering but does not significantly affect floral morphology [25,26].

Soybean (*Glycine max* [L.] Merr.) is an important global crop that provides protein and oil. Understanding of the processes that occur during soybean reproductive development and the identification of the genes responsible for developmental processes is important for soybean breeding. However, only a few reproductive-related genes have been identified in soybean, including *GmAP1*[27], *GmNMH7*[28], and *GmGAL1*[29]. Additionally, none of these soybean MADS-box genes were shown to alter floral morphology. As an attempt to understand flower developmental process in soybean and genetically modify soybean floral morphology for breeding applications, we identified 28 flower-enriched transcription factors in soybean through a microarray analysis [30]. Here, we report the functions of GmMADS28 which plays pivotal roles in the regulation of floral organ number and petal identity. The sterility caused by the ectopic expression of *GmMADS28* offers a promising approach to genetically produce new sterile material that could potentially be applied in crop hybrid breeding. Through the identification of more genes related with soybean flower, engineering soybean sterile materials applied in hybrid breeding programs will be possible.

## Results

### *GmMADS28* is a member of Class E MADS-box genes

**Figure 1:**
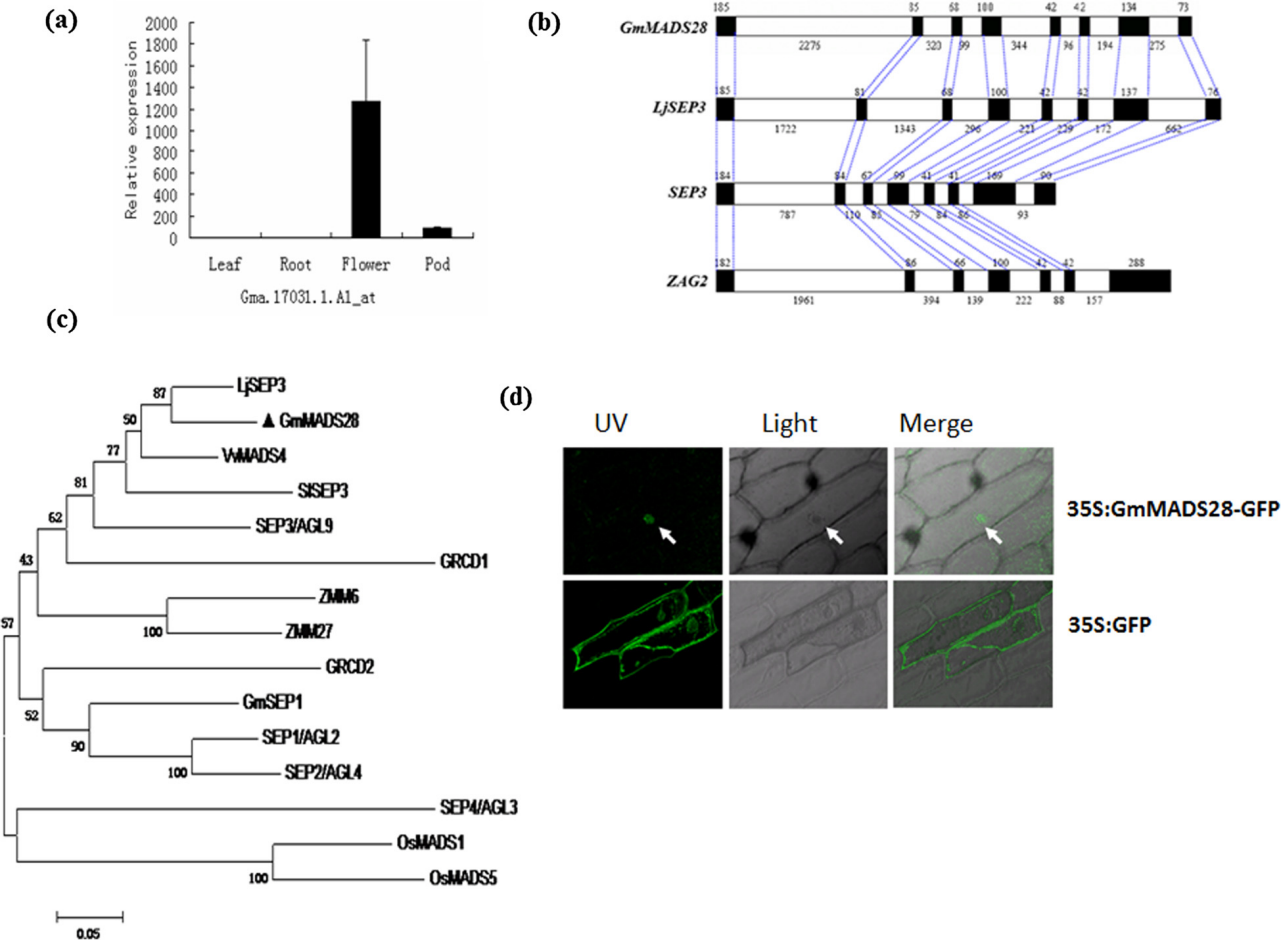
**Characterization of GmMADS28. (a)** Real-time qPCR analysis of *GmMADS28* in soybean leaves, roots, flowers and pods. **(b)** Exon-intron structures of *GmMADS28* and *Lotus japonicas LjSEP3*, Arabidopsis *SEP3* and maize *ZAG2*. The black and white boxes represent exons and introns, respectively. **(c)** Phylogenetic analysis of GmMADS28 and other plant SEP proteins. The neighbor-joining tree was constructed by MEGA 4.0 based on 1,000 bootstrap replicates. **(d)** Subcellular localization of GmMADS28-GFP fusion protein.

Among 28 transcription factor genes predominantly expressed in soybean flowers [30], a soybean homolog (Gma.17031.1.A1_at) of Arabidopsis *AGL9*/*SEP3* was selected for further analysis in the present study. The microarray data were confirmed by real-time qPCR (Figure 1a). We named this gene *GmMADS28* because it showed the highest similarity with Arabidopsis *SEP3*. First, we cloned the cDNA fragment of *GmMADS28* via RT-PCR from soybean flowers. Through RACE-PCR, we assembled and cloned the full-length cDNA of *GmMADS28*, which is 1,026 bp in length and contains an ORF of 732 bp. The sequence of *GmMADS28* has been deposited in GenBank/EMBL under accession number AJ878424.

Comparison of the *GmMADS28* cDNA and soybean genomic DNA sequences suggested that *GmMADS28* contains eight exons and seven introns. By comparison of the exon–intron structures of *GmMADS28* and other plant MADS-box genes, we found that *GmMADS28* and other plant *SEP* genes share the same numbers of exons and introns; each exon size is highly conserved, whereas the intron size is divergent (Figure 1b). The maize *AG* gene *ZAG2* has seven exons, though the sizes of the first six exons covering the MADS- and K-domains are similar to those of *GmMADS28*. These results suggested that sequence alteration in the MADS-box genes during genome duplications, particularly in the 3′ region, might have resulted in divergent subfamilies of plant MADS-box proteins.

The deduced GmMADS28 protein contains 243 amino acids and has a molecular mass of 27.9 kDa. Database searches suggested that GmMADS28 is homologous to Arabidopsis SEP3 (identity of 74% and similarity of 83%), SEP1 (identity of 59% and similarity of 71%), SEP2 (identity of 60% and similarity of 71%), and SEP4 (identity of 47%, and similarity of 64%). The alignment of GmMADS28 and the other plant MADS-box proteins showed that the amino acid sequences in the MADS domains were highly conserved. In addition to the MADS domain, the intergenic region (I region), K-box, and a C-terminal region (C region) are included in each MADS-box protein. Moreover, a potential phosphorylation site, RQVTF, for calmodulin-dependent protein kinases and three common sequences, the “SEP motif I”, “SEP/AGL6 motif”, and “SEP motif II” [31,32], were found in all the analyzed SEP proteins, including GmMADS28.

To address relationship of GmMADS28 to other plant SEP proteins, a neighbor-joining phylogenetic tree was constructed based on the alignment of amino acid sequences from the plant SEP proteins (Figure 1c). The tree showed that GmMADS28 was grouped into the plant SEP3 subfamily. In particular, GmMADS28 was closer to *Lotus japonicas* LjSEP3 and Arabidopsis SEP3. These results suggest that *GmMADS28* is an E gene; based on the tree and highest sequence identity between GmMADS28 and SEP3*.*

### GmMADS28 is localized in the nucleus

To assess the subcellular localization of GmMADS28, we fused the full-length ORF of *GmMADS28* to the green fluorescence protein (GFP) reporter gene under the control of the CaMV35S promoter to generate the construct 35S:GmMADS28-GFP. The construct and the empty vector were transformed into onion epidermal cells via *Agrobacterium*-mediated transformation. Confocal imaging of GFP fluorescence showed that the GmMADS28-GFP fusion protein was localized to the nucleus; in contrast, the GFP fluorescence was distributed throughout the cells when the cells were transformed with GFP alone (Figure 1d).

### Transcripts of *GmMADS28* accumulate predominantly in reproductive organs, especially in petals

**Figure 2:**
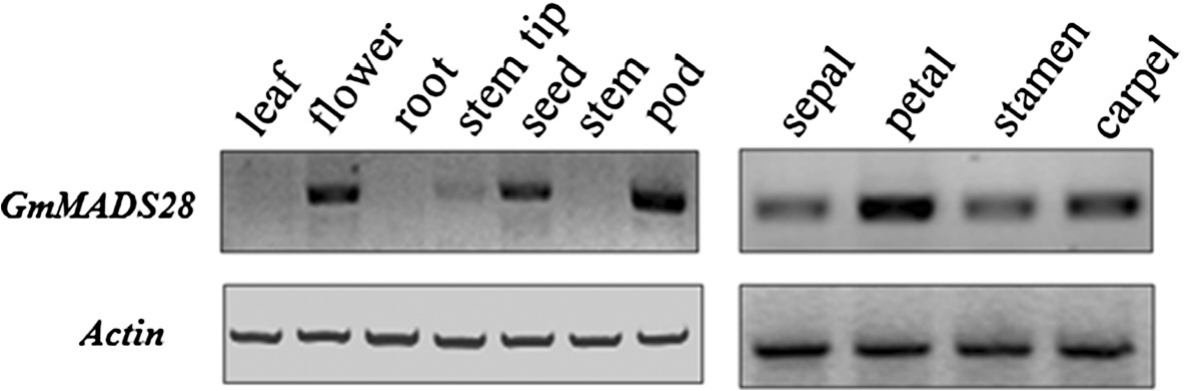
***GmMADS28 *expression in different tissues of soybean by semi-quantitative RT-PCR analysis.***Actin* gene was used as the reference gene.

The expression profiles of the *GmMADS28* in various soybean tissues were investigated by semi-quantitative RT-PCR. Various soybean tissues, including leaf, flower, root, shoot apex, seed, stem, and pod, were collected. The expression analysis showed that the *GmMADS28* mRNA was clearly detected in the reproductive organs, including the flower, seed, and pod, but not in the leaf or root (Figure 2). Weak expression of *GmMADS28* in the vegetative shoot apex was also found (Figure 2). To further analyze the expression of *GmMADS28* in flowers, we examined the expression of *GmMADS28* in the four whorl organs: sepal, petal, stamen, and carpel. As shown in Figure 2, *GmMADS28* was clearly detected in all four organs and showed the highest expression in petals.

Because *GmMADS28* was highly expressed in developing seeds (Figure 2), it is of interest to investigate whether *GmMADS28* is involved in soybean seed development. Thus, we performed a semi-quantitative RT-PCR assay to analyze the time-course expression pattern of *GmMADS28* during soybean seed development. The results showed that *GmMADS28* was highly expressed at 15 and 20 DAF (days after flowering) but was down-regulated thereafter, with the lowest level at 35 DAF; the expression of *GmMADS28* increased gradually after 40 DAF (Additional file 1: Figure S1). These results suggested that GmMADS28 may play some roles at the early and late stages of seed development.

### *In situ* localization of *GmMADS28 transcript*

**Figure 3:**
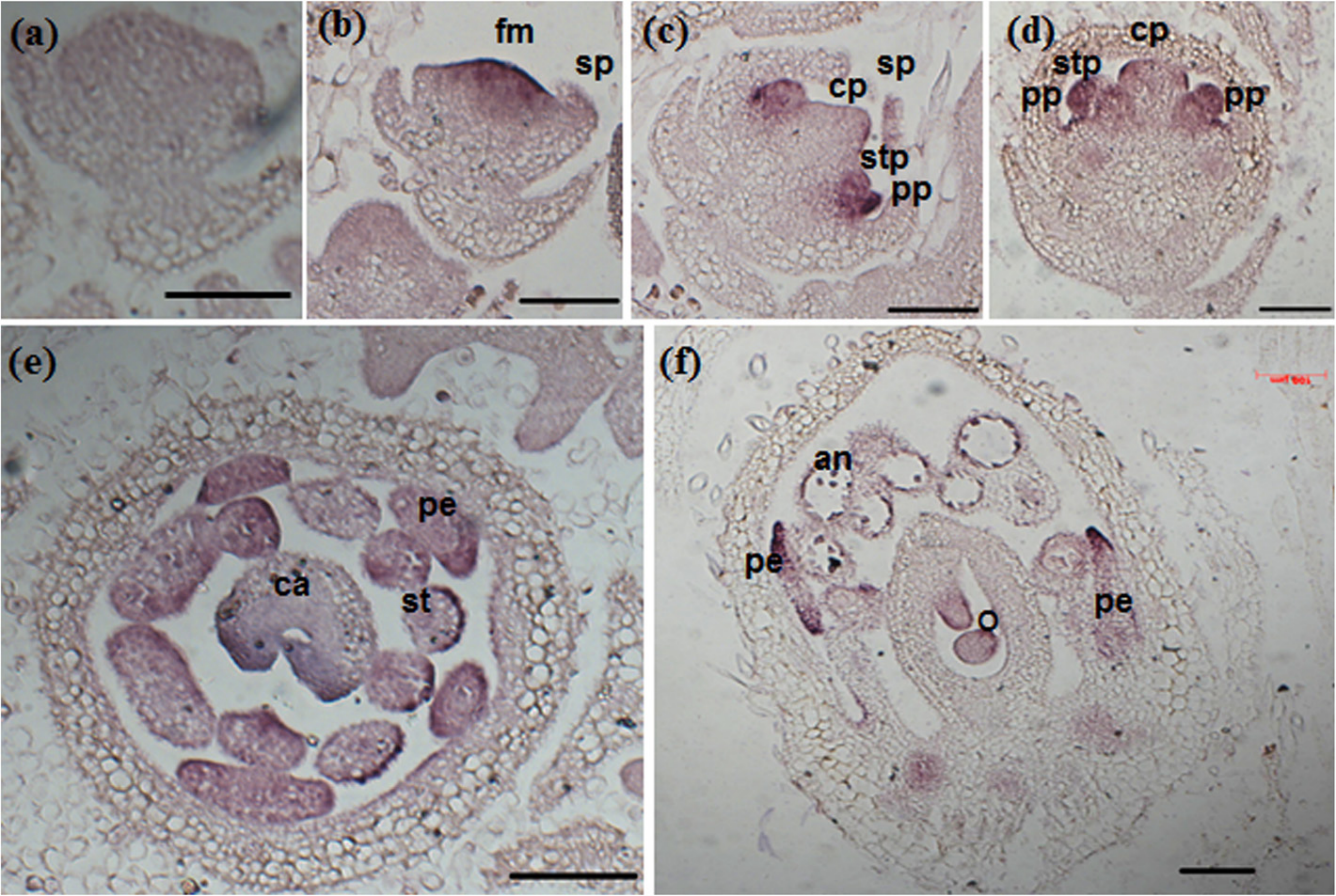
***In situ *hybridization of *GmMADS28 *in soybean developing flowers. (a-e)** The longitudinal sections of flowers at differential developmental stages hybridized with an antisense **(b-f)** or sense **(a)** probe. **(e)** The cross section of flowers at the mature stage. fm, floral meristem; sp, sepal primordium; pp, petal primordium; stp, stamen primordium; cp, carpel primordium; st, stamen; pe, petal; ca, carpel; an, anther; o, ovule. Bars: 100 μm.

To further analyze the spatial distribution of *GmMADS28*, mRNA *in situ* hybridization was employed to detect floral mRNA expression. In the early stages of flower development, transcripts of *GmMADS28* were localized in the floral meristem (Figure 3b), and in the stamen and petal primordia (Figure 3c-d). In the late stages of flower development, transcripts of *GmMADS28* were detected in the stamens, petals, and ovules (Figure 3e-f).

### GmMADS28 promotes early flowering

**Figure 4:**
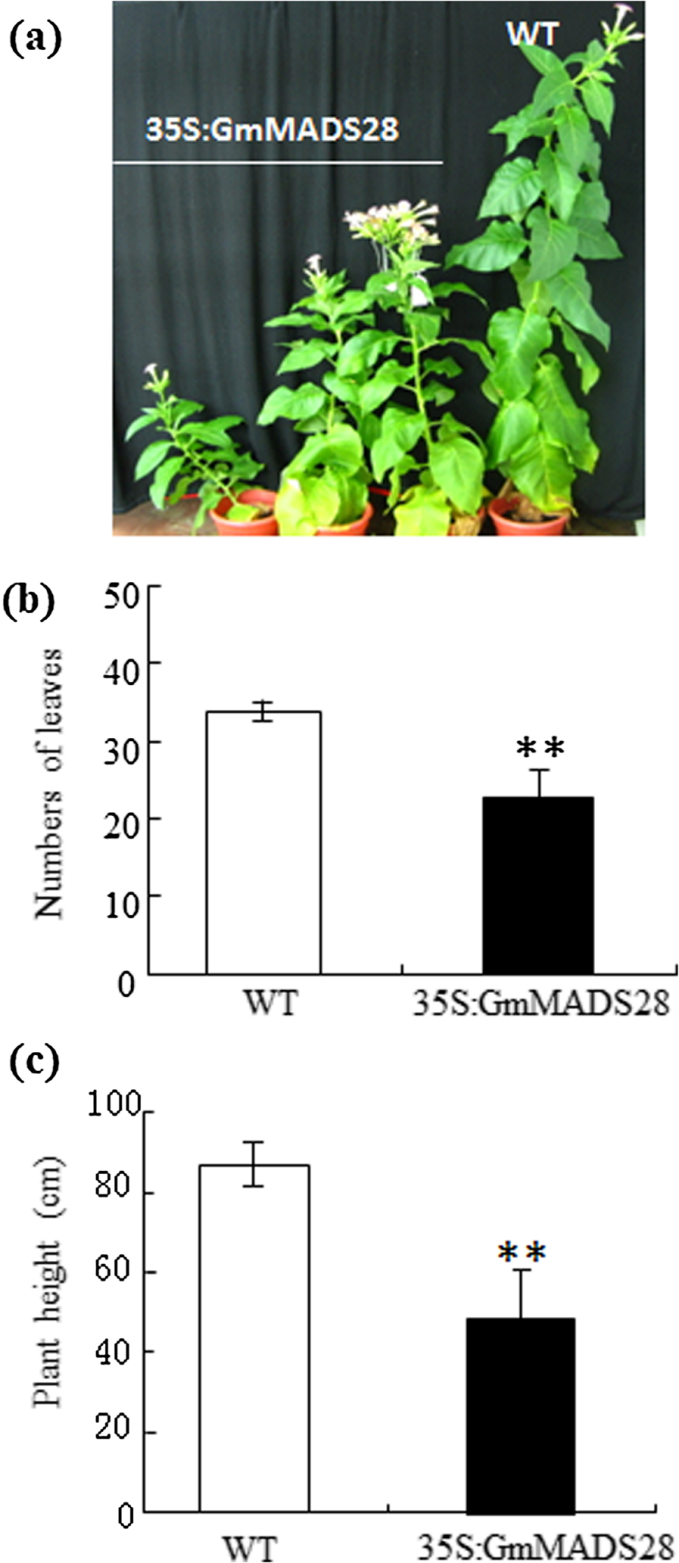
**Ectopic expression of *GmMADS28 *promotes early flowering. (a)** Selected transgenic plants at flowering stage. **(b)** The number of leaves of wild-type and 35S:GmMADS28 plants when flowering. **(c)** The plant height of wild-type and 35S:GmMADS28 plants when flowering. The error bars represent SD (n = 10).

To analyze the biological role of GmMADS28, we ectopically expressed the *GmMADS28* gene in tobacco (Figure 4a) and obtained 13 independent transgenic lines overexpressing *GmMADS28* confirmed by RT-PCR analysis (Additional file 2: Figure S2). We found that all positive 35S:GmMADS28 plants flowered significantly earlier than the wild-type plants. As the plants have a fixed number of leaves when flowering under the same environment, a comparison of the flowering time was evaluated by the leaf number. The 35S:GmMADS28 plants produced an average of only 22 leaves when flowering, whereas the wild-type plants produced approximately 34 leaves (Figure 4b). The 35S:GmMADS28 plants also exhibited reduced plant height compared to the wild-type plants (Figure 4c).

### Ectopic expression of *GmMADS28* affects morphology of floral organs

**Figure 5:**
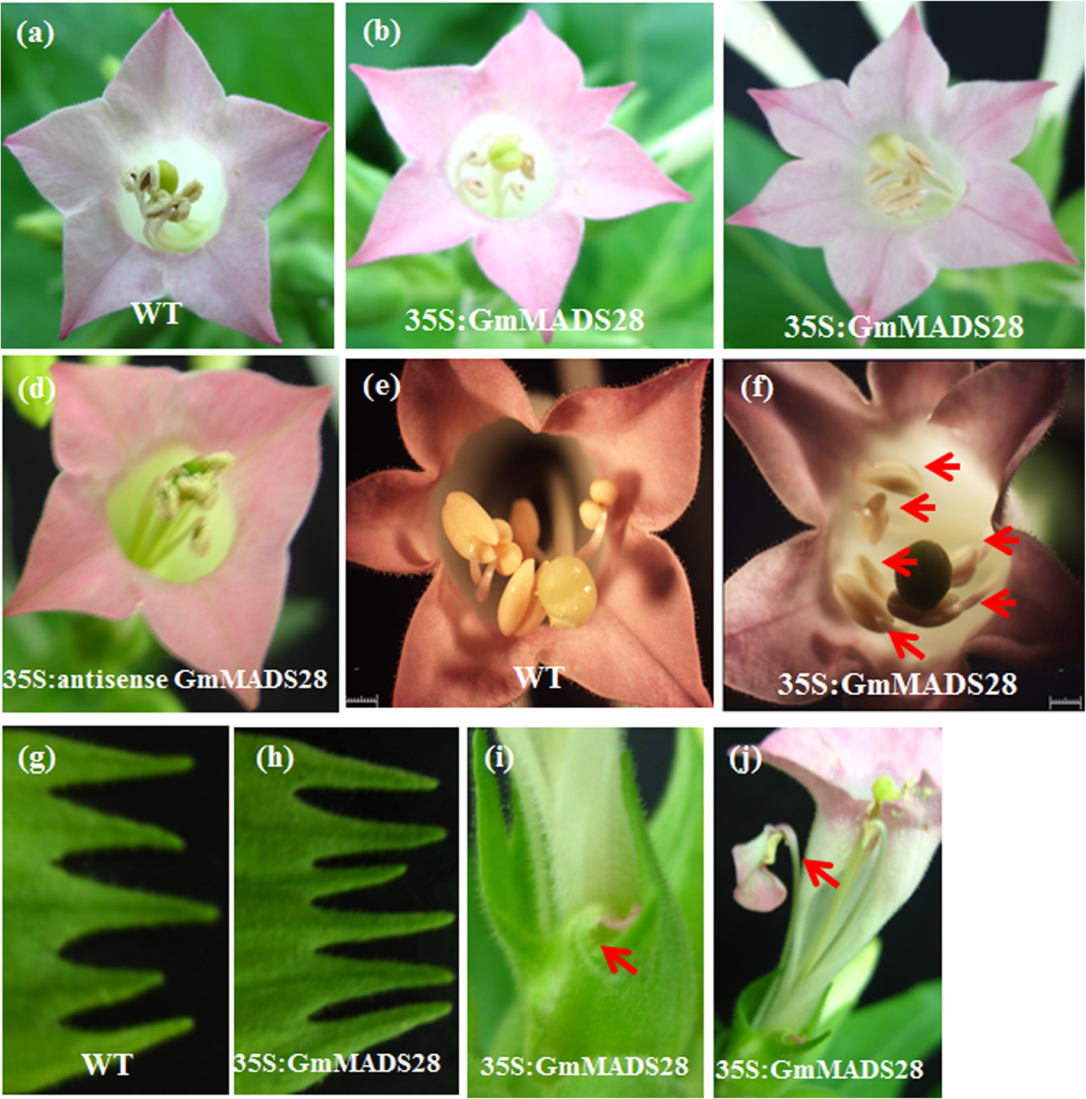
**Phenotypes of 35S:GmMADS28 at the reproductive stage. (a, e)** WT flower with five petals and stamens. **(b, c, f)** 35S:GmMADS28 flowers with six petals and stamens. **(d)** 35S:antisense GmMADS28 flower with four petals. **(g)** WT with five sepals. **(h)** 35S:GmMADS28 with six sepals. **(i)** 35S:GmMADS28 converts sepal to petal. **(j)** 35S:GmMADS28 converts stamen to petal. The arrows in **(f)** indicate the stamens. Bars: 1 mm in **(e-f)**.

The *GmMADS28* over-expression lines exhibited remarkable morphological alterations in the floral organs. In wild-type plants, the number of petals, stamens, and sepals is five, but six 35S:GmMADS28 lines displayed six for each organ (Figure 5a-c, e-h). Interestingly, antisense expression of *GmMADS28* in transgenic tobacco reduces the numbers of petals from five to four but the numbers of other three whorl organs are still five (Figure 5d). Besides, four 35S:GmMADS28 lines converted the stamens into petals and two converted the sepals into petals. The ectopic expression of *GmMADS28* could convert the sepals and stamens into petals (Figure 5i, j), implying the regulation by GmMADS28 in petal identity.

**Figure 6:**
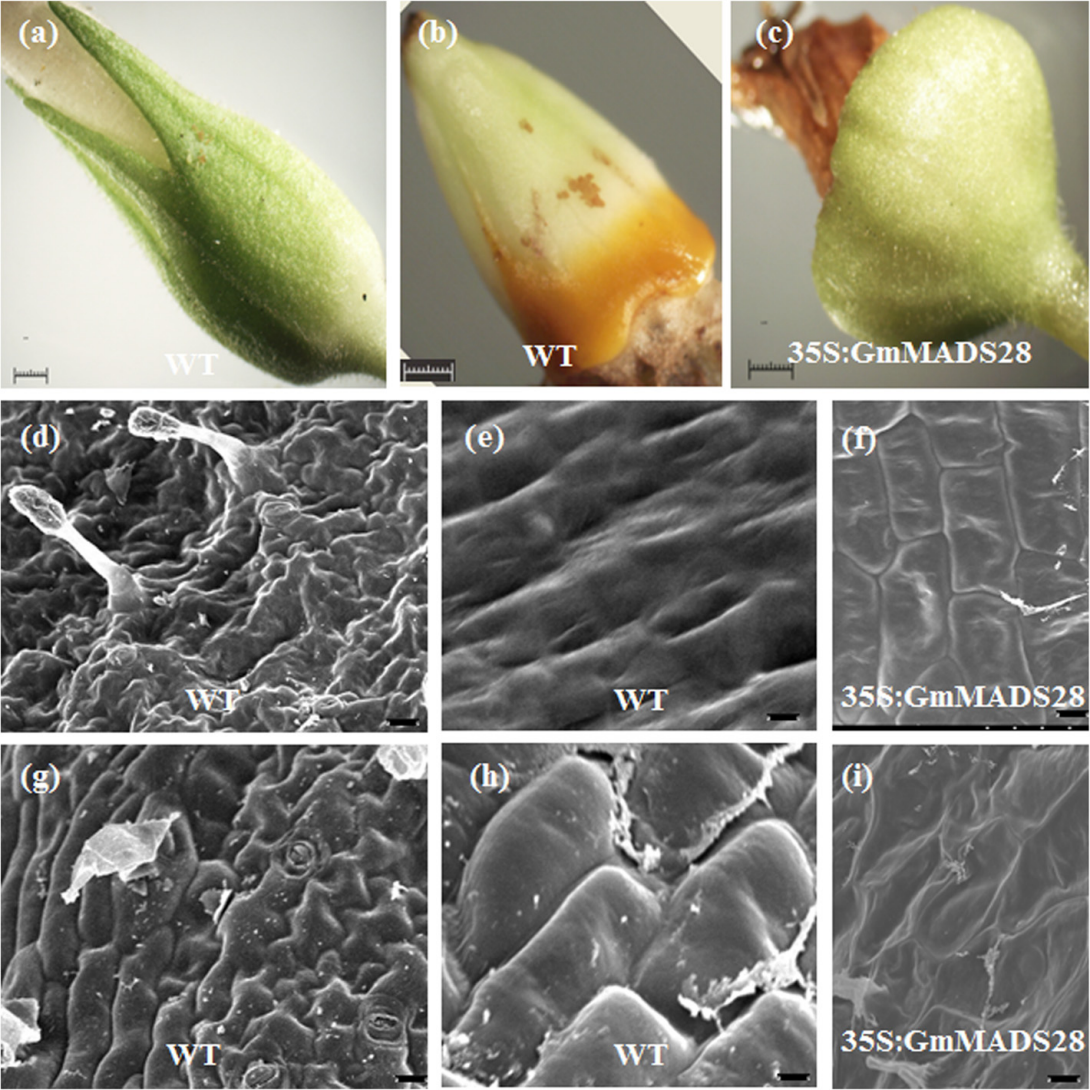
**35S:GmMADS28 develops the carpel-like sepals. (a)** WT sepal. **(b)** WT carpel. **(c)** 35S:GmMADS28 sepal. The inner **(d, e, f)** and outer **(g, h, i)** epidermal cells of WT sepals and carpels and 35S:GmMADS28 carpel-like sepals were observed through scanning electron microscopy (SEM) analysis. Bars: **(a-c)** 1 mm; **(d, f, g, i)** 10 μm; **(e)** 5 μm; **(h)** 2 μm.

Moreover, the sepals of the three 35S:GmMADS28 transgenic lines were more similar to the carpels than to the sepals of the wild-type plant (Figure 6a-c). The inner and outer epidermal cells of the tobacco sepal, which are characterized by the presence of globular-tipped trichomes, stomata, and irregularly shaped cells, were used for scanning electron microscopy (SEM) analysis (Figure 6d, g). However, similar to the wild-type carpel, the inner and outer epidermal cells in the 35S:GmMADS28 sepals were regular rectangular-shaped cells (Figure 6e-f, h-i). These results suggest that GmMADS28 might involve in the carpel identity.

### 35S:GmMADS28 plants are sterile due to physical alterations in floral structure

**Figure 7:**
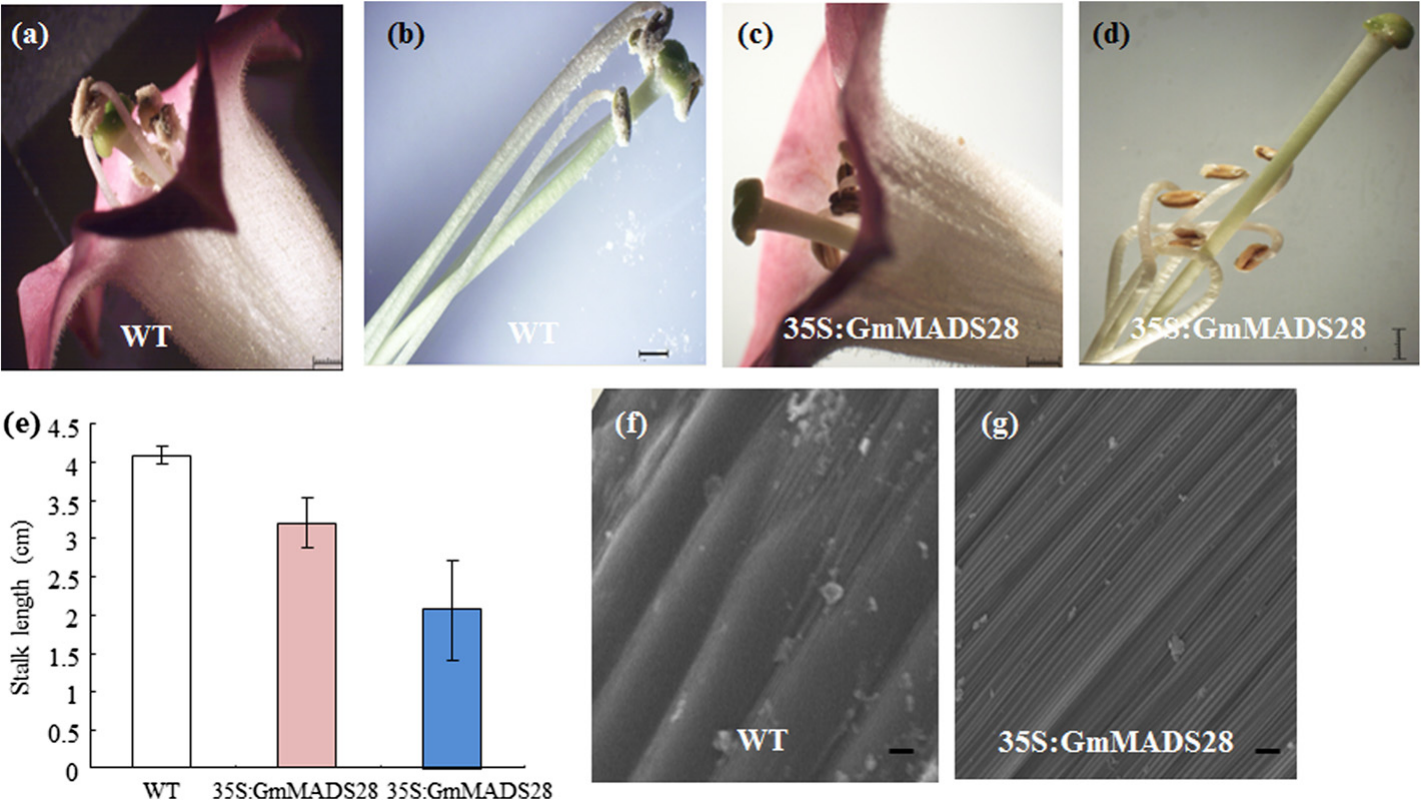
**35S:GmMADS28 develops the shortened and curly filaments. (a, b)** WT stamens can touch the stigma. **(c, d)** 35S:GmMADS28 stamens are shortened and curly and cannot touch the stigma. **(e)** Comparison of filament length of WT and 35S:GmMADS28. The blue column indicates the length of 35S:GmMADS28 curly filaments while the red column the straightened filaments. The error bars represent SD (n = 25). **(f, g)** The epidermal cells of WT and 35S:GmMADS28 filaments were analyzed by SEM. Bars: **(a-d)** 1 mm, **(f)** 3 μm and **(g)** 5 μm.

The 35S:GmMADS28 plants were sterile. To investigate the factors causing sterility, we examined the morphological structure of the floral organs. As shown in Figure 7, eleven 35S:GmMADS28 lines showed shortened and curly filaments, with the filament being shorter than the pistil (Figure 7c, d). The lengths of the filaments were measured before and after stretching. The filament length of 35S:GmMADS28 was significantly shorter than that of wild-type (Figure 7e), and the stamens could not reach the stigma due to such a shortened filament. The SEM analysis on the outer epidermal cells of the filaments was performed to analyze the reason for the shortened filaments. Compared to the wild-type filaments, which contain normal cells (Figure 7f), the 35S:GmMADS28 filaments only have cellular wrinkles, which may lead to curly filaments (Figure 7g).

**Figure 8:**
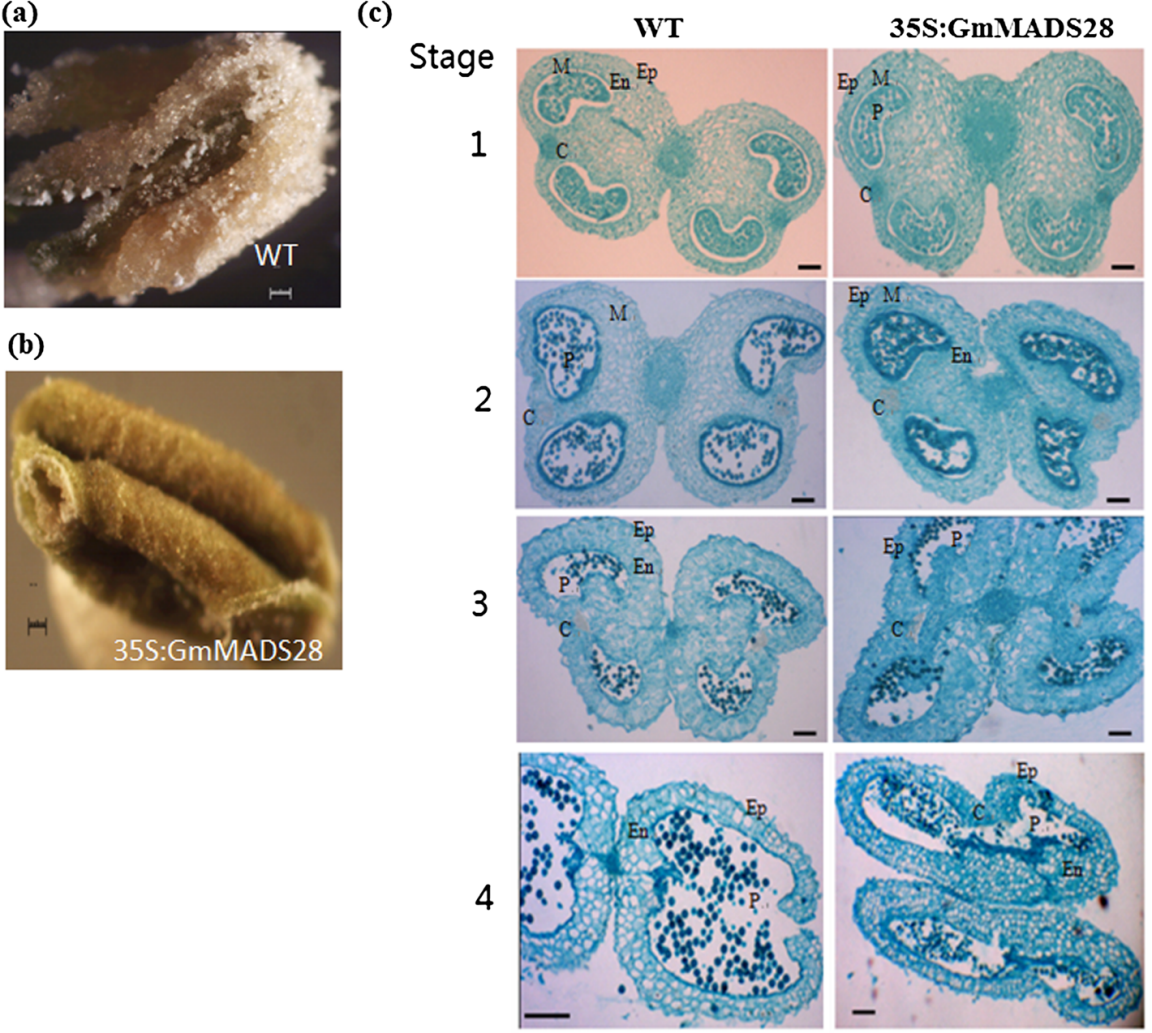
**35S:GmMADS28 fails to release the pollens. (a)** WT anthers with pollens. **(b)** 35S:GmMADS28 anthers without pollens covering. **(c)** Cellular comparison of anther development between WT and 35S:GmMADS28. The anther dehiscence was observed at stage 4 of WT anthers (indicated by red arrow). Ep, epidermic cell; M, middle layer cells; En, endothecium cells; P, pollens; C, parenchyma cells. Bars: **(a-c)** 100 μm.

In addition to shorter filaments, most of the 35S:GmMADS28 anthers cannot split normally, and the pollens cannot be released (Figure 8a-b). To reveal the formation of the unopened anthers, the anthers from 35S:GmMADS28 and wild-type plants were collected at four flower development stages, and the anther morphology of paraffin sections was compared using a microscope. It was found that there were no significant differences between 35S:GmMADS28 and wild-type at the beginning stages of anther maturation (Figure 8c, stages 1 to 2). However, although pollenss were released with the maturation of the wild type (WT) anthers (Figure 8c, stages 3 to 4), the 35S:GmMADS28 anthers could not split (Figure 8c, stages 3 to 4) and the pollens were not released.

### GmMADS28 activates the expression of *SOC1*, *LEAFY, AGL8/FUL* and *DEF*

**Figure 9:**
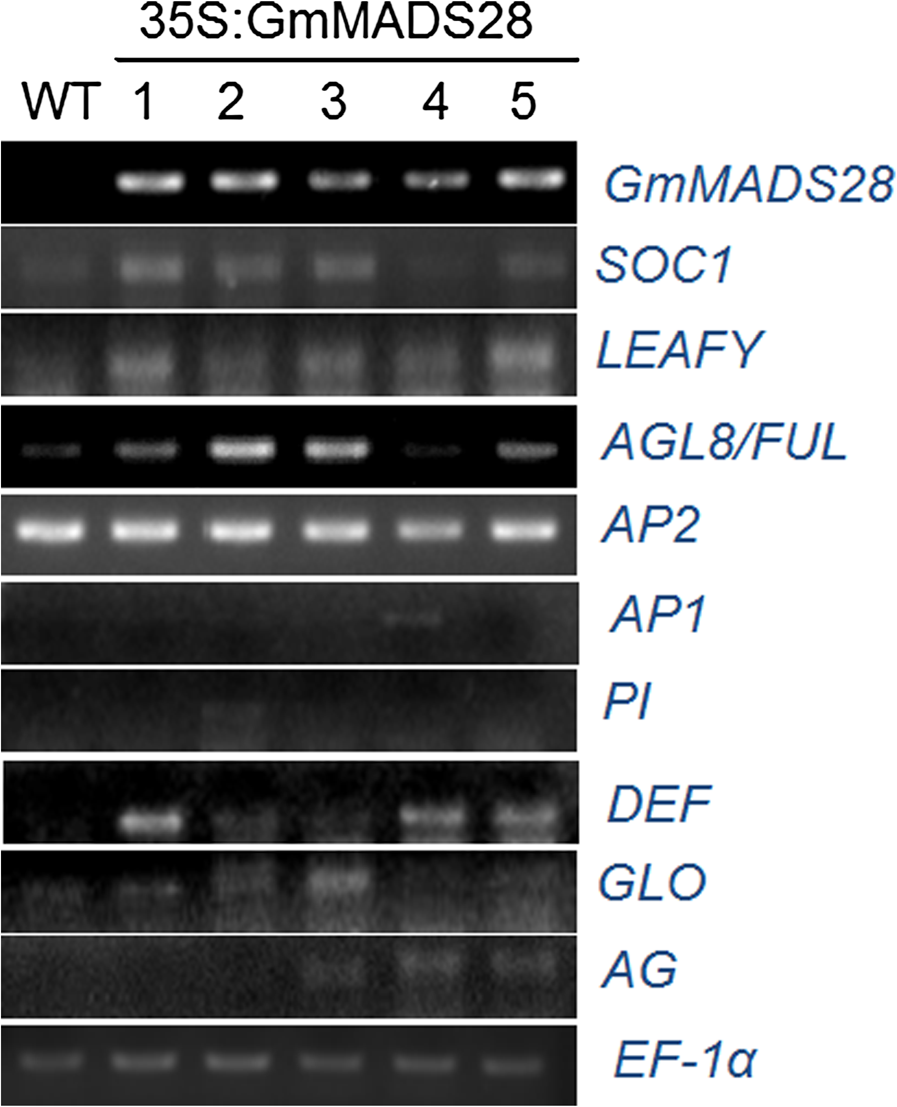
**Expression of 9 MADS-box genes in 35:GmMADS28 leaves.** Semi-quantitative RT-PCR analysis showed the expression of 9 MADS-box genes involved in flowering time (*SOC1*, *LFY*) and floral organ development in tobacco leaves. These tobacco genes are the closest homologues of the corresponding Arabidopsis genes. The accession numbers for these genes are shown in Additional file 4: Table S1.

Based on the function of GmMADS28 revealed by its constitutive expression, we studied the expression of 9 genes involved in flowering time or organ identity in the 35S:GmMADS28 leaves. It was observed that the tobacco homologs of the flowering time genes *SOC1* and *LEAFY*, A gene *AGL8/FUL*, and B gene *DEF* were more accumulated in 35S:GmMADS28 compared to wild-type leaves (Figure 9), suggesting that GmMADS28 might directly regulate the expression of these genes, thereby controlling flowering time and petal identity.

### GmMADS28 is involved in the conversion of stamens to petals in a soybean mutant

**Figure 10:**
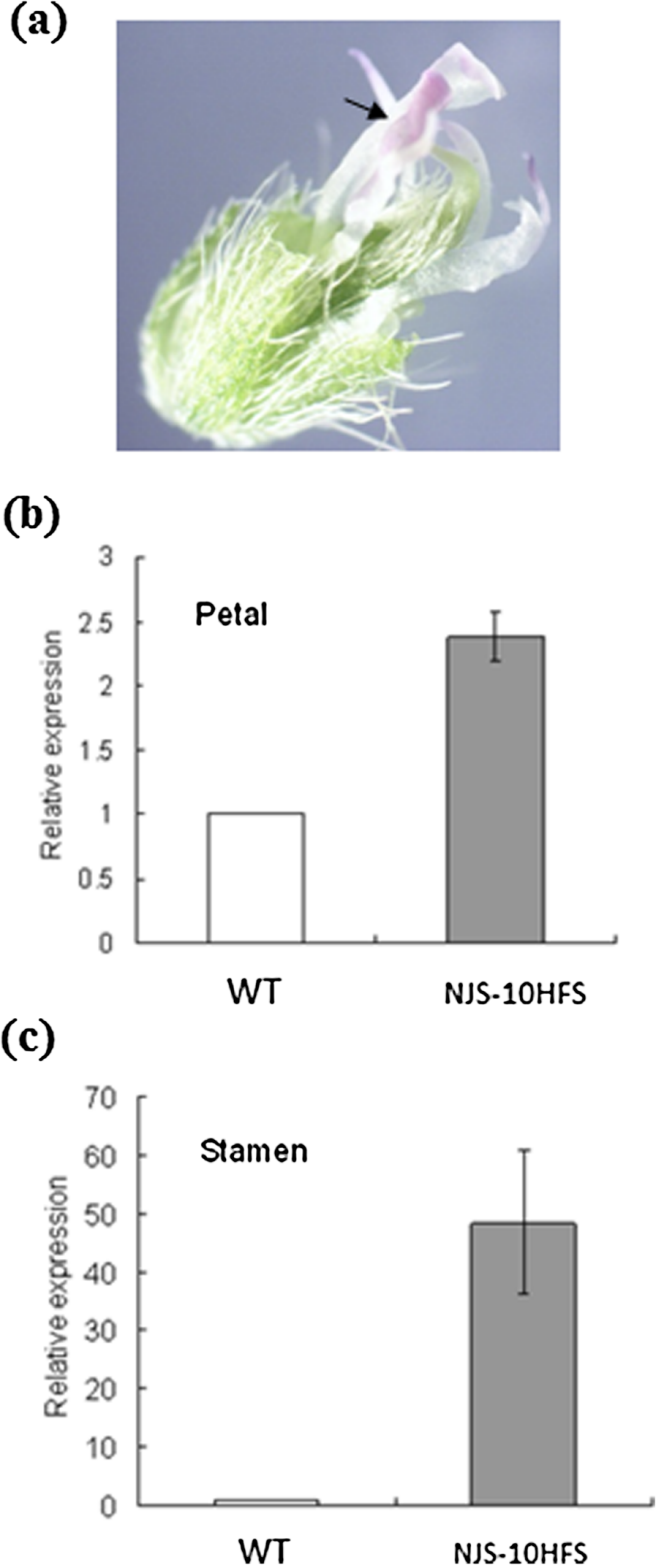
**Expression of *GmMADS28 *in soybean mutant NJS-10Hfs. (a)** The flower without petals of the mutant. The arrow indicates the conversion of the stamens into petals. **(b)** Real-time qPCR analysis of *GmMADS28* expression in petals of the mutant NJS-10Hfs and those of WT. **(c)** Real-time qPCR analysis of *GmMADS28* expression in stamens of the mutant NJS-10Hfs and those of WT. The error bars represent SD based on three replicates.

The 35S:GmMADS28 transgenic tobacco plants exhibited the conversion of stamens to petals, which is also the phenotype of soybean mutant NJS-10Hfs [30,33] (Figure 10a). Compared to wild-type soybean stamens, the top areas of most stamens are converted to petal-like structure in NJS-10Hfs. Therefore, it is of interest to investigate the expression of *GmMADS28* in the flower organs of this mutant. We found that expression of *GmMADS28* in the stamens and petals in NJS-10Hfs was higher than in wild-type NJS-10Hff (Figure 10b, c). The expression of *GmMADS28* was particularly higher in the mutant stamens (~50 folds) compared to wild-type (Figure 10c), suggesting that the up-regulation of *GmMADS28* may play a critical role in the conversion of stamens to petals in this soybean mutant.

### GmMADS28 interacts with soybean homologs of SOC1, AP1, and AGL8/FUL

As MADS-box proteins can form different complexes with different functions during floral development, we screened for interacting partners of GmMADS28 by yeast two hybrid assay using a cDNA library prepared from soybean flowers. A total of five proteins (GmSIP1 ~ GmSIP5), including soybean homologs of AGL8/FUL, SOC1, and AP1 proteins, an ENTH/VHS family protein [34], and an unknown protein, were shown to interact with GmMADS28 (Table 1, Additional file 3: Figure S3). These proteins may form complexes with GmMADS28 and function in the regulation of flowering time and floral development.

**Table 1:** Soybean proteins interacting with GmMADS28

**Name**	**Gene ID**	**Annotation**
GmSIP1	Glyma11g36110	ENTH/VHS family protein
GmSIP2	Glyma05g32820	Unknown protein
GmSIP3	Glyma04g31800	AGL8/FUL
GmSIP4	Glyma18g45780	SOC1
GmSIP5	Glyma16g13070	APETALA1

## Discussion

Despite their similar sequences, plant *SEP3* genes vary in their expression patterns. *SEP3* is expressed throughout flower development, from the floral meristem to the fully developed floral organs [35]. The *SEP3* genes are expressed in the inner three floral whorls of all species, with the exception of Aranda *AdOM1*, which is not expressed in stamens and carpels [36]. *GmMADS28* also showed a weak expression in the soybean shoot apices in addition to flowers and seeds, indicating that GmMADS28 might also participate in biological events in the shoot apices. In fact, Gerbera *GhGRCD2* is also expressed in vegetative tissues [11]. Although *GmMADS28* was expressed in all the reproductive organs analyzed, including the four whorl organs, it showed the highest expression level in petals, suggesting that GmMADS28 might play an important role in general flower development but is particularly involved in controlling the identity and development of soybean petals.

To further explore the GmMADS28 function, we ectopically expressed the *GmMADS28* gene in tobacco and found that GmMADS28 promotes early flowering. Many studies have shown that the overexpression of *SEP3*-like gene leads to early flowering [23,25,37,38]. In Arabidopsis, the SEP3 protein is potentially incorporated into complexes with proteins involved in flowering time, such as SOC1, AGL24, SVP, and AGL15 [16]. The Arabidopsis *SOC1*-like genes AGL42, AGL71, and AGL72 promote flowering in the shoot apical and axillary meristems [39]. Interestingly, our data showed that GmMADS28 activates the expression of the tobacco homologs of *LEAFY* and *SOC1*, implying that the activated expression of these two genes may lead to early flowering. However, *SOC1* was shown to be directly down-regulated by SEP3 in Arabidopsis [18]. These contrasting observations may be due to the different plant systems. Enhanced early flowering was also observed in Arabidopsis plants over-expressing both *SEP3* and *AP1* genes compared to plants over-expressing *AP1* alone [22]. Similarly, we observed that the ectopically expression of soybean *GmAP1* also promotes early flowering in Arabidopsis [27]. These results suggest that GmMADS28 and AP1 may be incorporated into the MADS-box protein complex to cooperatively regulate flowering time, which is also supported by the interaction of the GmMADS28 and AP1 proteins by yeast two-hybrid assay.

In Arabidopsis, *SEP* genes are involved in the development of all four-whorl floral organs and that constitutive expression of *SEP3* promotes the homeotic transformation of sepals and inflorescence meristems into carpelloid structures, including ovules [23]. In the present study, a number of floral developmental alterations were observed in *GmMADS28* transgenic plants. Interestingly, not all transgenic plants showed the same/similar phenotypes, which is likely because that *GmMADS28* as an E-type gene plays a wide role in all most aspects of floral development and different expression level and genome insertion location may influence the phenotypes. Even in one plant, not all the flowers showed the same phenotypes. For example, in the lines with increased number of floral organs, about half of the flowers altered organ number, while in the sterile lines, all the flowers are sterile.

Ectopic expression of *GmMADS28* promoted the development of extra set of floral organs in transgenic tobacco, implying the critical and positive role of GmMADS28 in the formation of floral organs. Interestingly, the antisense expression of *GmMADS28* decreased the petal number from five to four, which might have resulted from the decrease of native *SEP3* expression in the tobacco flowers. In contrast, Zhao et al. [24] found that the ectopic expression of a wheat *SEP*-like gene, *TaMADS1*, decreased the petal and stamen number, suggesting that different SEP proteins might play diverse roles in the regulation of floral development. Our data suggest that GmMADS28 is involved in the formation of four-whorl organs and is particularly required for normal petal and stamen development. The A, B, C, and SEP proteins likely act as multimeric complexes to activate downstream genes [8,10,22,40]. The expression analysis of several ABC genes in *35:GmMADS28* leaves suggests that the ectopic expression of *GmMADS28* itself could activate the expression of the *DEF* and *AGL8/FUL* genes. The multimeric complexes formed with activated ABC proteins and GmMADS28 may promote the production of extra organs in the transgenic plants.

The ectopic expression of *GmMADS28* mediated the conversion of stamens and sepals into petals in transgenic tobacco. In our previous study, we identified a soybean mutant [33] with a phenotype similar to 35S:GmMADS28, with the conversation of stamens into petals. Interestingly, the expression of *GmMADS28* in the stamens of the soybean mutant was significantly increased compared to the wild-type plants, and the mRNA *in situ* localization analysis proved the predominant expression of *GmMADS28* in the petal meristem of soybean. These results indicate that GmMADS28 plays a critical and positive role on the B function. Our data showed that ectopic expression of *GmMADS28* activates the expression of B gene *DEF*[41] in tobacco leaves, suggesting that the ectopic expression of *GmMADS28* can confer B function. Moreover, an A class gene, *AGL8/FUL*, was activated by GmMADS28. Recently, it was found that FUL-like genes also control flowering time and petal identity in opium poppy [42].

Carpel-like sepals were observed in some of the 35S:GmMADS28 plants and the constitutive expression of *SEP3* resulted in the homeotic transformation of sepals and inflorescence meristems into carpelloid structures in Arabidopsis [23]. It has been described that both SEP3 and AG are required for functional C activity [10,40] and that SEP3 could ectopically activate *AG*[23] in 35S:SEP3 leaves. However, unlike Arabidopsis, the expression of *AG* was not significantly regulated by GmMADS28 in our study, suggesting that GmMADS28 might have an AG-independent role in the formation of carpel-like sepals.

Controlling male fertility is an important goal for breeding hybrid crops, and male sterility is associated with the failure of pollination in addition to the lack of viable pollen or pollen activity. We observed that the 35S:GmMADS28 plants are sterile, which was caused by two major factors, shortened and curly filaments and the failure of pollen release, which are new findings of SEP3 function. At the beginning stages of anther maturation, there were no significant differences between 35S:GmMADS28 and the wild type plants. The epidermal cells absorb water from the endothecium cells due to transpiration during anther maturation, which may produce tension in the un-thickened, adjacent regions of the two pollen sacs. For normal anthers, further water loss during maturation causes the parenchyma tissue between the pollen sacs to rupture easily under the tension developed by the endothecium, thus releasing the pollen. For 35S:GmMADS28 anthers, it is hypothesized that the endothecium cells are thickened and the shriveled connection of the cellulose can’t pull out the easy-rupture region, the tension was not sufficient to split the anthers; therefore, the pollen grains can’t be released.

Unlike the role of GmMADS28, the overexpression of the *SEP3* genes from Arabidopsis, tobacco, and rice in wild-type Arabidopsis or tobacco promotes early flowering but does not significantly affect floral morphology [22,25,26]. These results suggest that plant SEP3 proteins have common functions in the regulation of flowering time. In this study, we observed the roles of SEP3 proteins in the regulation of floral organ number and petal identity. More importantly, the sterility caused by the ectopic expression of *GmMADS28* offers a promising way to genetically produce new sterile material that could potentially be applied in the hybrid breeding of crops like soybean.

## Conclusion

The molecular regulations on reproductive development in soybean are largely unknown. Additionally, controlling the fertility is an important goal in plant hybrid breeding but is difficult in some crops including soybean. Through microarray analysis, a flower-enriched gene *GmMADS28* encoding a MADS-box transcription factor was cloned from soybean. *GmMADS28* belongs to E-type gene and may play a wide role in reproductive development. It was observed that constitutive expression of *GmMADS28* in tobacco caused a number of reproductive development changes, including early flowering, conversion of stamens and sepals to petals, increased numbers of sepal, petal and stamens and carpel-like sepals. In particular, ectopic expression of *GmMADS28* caused sterility due to the shortened and curly stalks and the failure of pollen release from the anthers. *GmMADS28* is thus a potential target gene for engineering the male sterile plants. Moreover, GmMADS28 was found to activate the tobacco homologs of *SOC1*, *LEAFY*, *AGL8/FUL* and *DEF* and interact with soybean homologs of SOC1, AP1 and AGL8/FUL proteins, which provides the new clues in understanding the functional mechanisms for E-type proteins in plant reproductive development.

## Methods

### Plant materials

The soybean (*Glycine max* L. Merr. cv Jackson) seeds used in this study were obtained from National Center for Soybean Improvement, Nanjing Agricultural University, China, and germinated in the experimental field of Nanjing Agricultural University. Mature leaves (fully expanded), roots, flowers (including flower buds and mature flowers), and pods at 20 days after flowering (DAF) were collected for the real-time RT-PCR analysis. Mature leaves (fully expanded), roots, stems (including internodes and nodes), and shoot apices were collected separately at the stage of the sixth expanding true leaf for semi-quantitative RT-PCR. At the flowering stage, mixed flowers (including flower buds and mature flowers), sepals, petals, stamens, and carpels were collected. Seeds were collected at 15, 20, 25, 35, 40, 45, and 50 days after flowering.

### Cloning of *GmMADS28*

To amplify a fragment (Probe ID: Gma.17031.1.A1_at) of *GmMADS28*, we first obtained the expressed sequence tag (EST) sequence for Gma.17031.1.A1_at by searching the Affymetrix website (https://www.affymetrix.com/) using the NetAffx tool. A pair of primers (forward, 5′-GAGATGGGAAGGGGAAGAGT-3; reverse, 5′-ACAAATTGGATATCATCCTG-3′) was synthesized for RT-PCR and used with flower cDNA as the template. The PCR conditions were as follows: 0.5 μl flower cDNA was amplified in a 25 μl volume containing 2.5 μl PCR buffer with MgCl_2_, 0.5 μl 20 mM dNTPs, 1 μl Tag polymerase, and 0.5 μl each specific primer (25 mM). The PCR amplification was performed using a DNA amplification machine (MJ, USA) with an initial denaturation at 94°C for 5 min, 33 cycles of 94°C for 30s, 55°C for 50s, and 72°C for 1 min, and a final 72°C for 10 min. The PCR products were gel-purified, cloned into the pGEM-T vector (Promega, USA), and sequenced (Invitrogen, Shanghai, China). Rapid amplification of cDNAs (RACE) was employed with SMART RACE technology (Clontech, USA) to obtain the full-length cDNA for *GmMADS28*. To amplify the full-length cDNA of *GmMADS28*, the following pair of primers was used for RT-PCR: forward, 5′-GAGATGGGAAGGGGAAGAGT-3′, and reverse, 5′-CAAGGAAGAGGCTAGCTAGG-3′.

### Real-time RT-PCR

Real-time qPCR was performed in an optical 96-well plate using a BIO-RAD iQ5 real-time PCR system (BIO-RAD, USA), as previously described [30]. The soybean *Actin* gene (GenBank accession No. V00450) served as a reference gene. The threshold cycle (Ct) values of the triplicate PCRs were averaged, and the relative quantification of the transcript levels was performed using the comparative Ct method. To verify the microarray data for *GmMADS28* expression, the relative quantification relates the PCR signal of the target transcript in the roots, flower mixtures, or pods to that in the leaves. The fold change was determined by the following formula: fold change = 2^−ΔΔCt^, where ΔΔCt = (Ct_target gene_ − Ct_*Actin*_)_flowers,__roots, or pods_ − (Ct_target gene_ − Ct_*Actin*_)_leaves_. For the analysis of *GmMADS28* expression in the soybean mutant, the relative quantification relates the PCR signal of the target transcript in the organs of the mutant to that of the wild-type plant. The fold change was determined by the following formula: fold change = 2^− ΔΔCt^, where ΔΔCt = (Ct_target gene_ − Ct_*Actin*_)_mutant_ − (Ct_target gene_ − Ct_*Actin*_)_wild − type plant_.

### Semi-quantitative RT-PCR

Semi-quantitative RT-PCR was performed as previously described [30]. As a control, a 683 bp PCR fragment of the constitutively expressed soybean *Actin* gene was amplified. The primers for *GmMADS28* were as follows: sense, 5′-GAGATGGGAAGGGGAAGAGT-3′, and reverse, 5′-CAAGGAAGAGGCTAGCTAGG-3′. For the RT-PCR analysis of nine tobacco MADS-box genes, total RNA was prepared from the leaves of the wild type and five 35S:GmMADS28 plants. The tobacco *EF-1α* gene was used as an internal control. The primers for each tobacco MADS-box gene are shown in Additional file 4: Table S1.

### RNA *in situ* hybridization

Longitudinal and cross sections of soybean flowers were prepared as previously described [27,43]. RNA antisense and sense probes were generated from a 281 bp fragment of the 3′ region of the *GmMADS28* cDNA labeled with digoxigenin. The RNA *in situ* hybridization was performed as previously described [27].

### Subcellular localization

To produce the *GmMADS28-GFP* construct, *GmMADS28* was fused in-frame to the 5′ terminus of the green fluorescent protein (GFP) reporter gene under the control of the CaMV35S promoter. The following primers was used for the *GmMADS28-GFP* construction: 5′-GAGATGGGAAGGGGAAGAGT-3′ (forward), and 5′-CAAGGAAGAGGCTAGCTAGG-3′ (reverse). The construct was then used in *Agrobacterium*-mediated transient expression in onion epidermal cells. The cells were examined by confocal laser-scanning microscopy (Leica TCS SP2, Mannheim, Germany).

### Ectopic expression in tobacco

To address the function of *GmMADS28*, the sense and antisense *GmMADS28* sequence were cloned into the plant binary vector pBI121. The recombinant plasmids were used for tobacco (*Nicotianatabacum* cv. SamSun) transformation via the leaf disk transformation method [44].

### Cell morphological analysis

Tobacco anthers were collected at flower lengths of 0.2, 0.5, 1, and 3 cm, fixed with Carnoy’s Fluid (ethanol: glacial acetic acid = 3:1) for up to 24 hours at room temperature, and then preserved in 70% ethanol at 4°C. After embedding in paraffin, the blocks were trimmed as necessary and cut into 8–10 μm sections using a LEICA RM2135 microtome. These sections were stained with safranin and stained again with fast green. After mounting the sections onto slides, the tissues were observed and photographed with a microscope (Leica DMLB).

### Yeast two-hybrid assay

As the full-length GmMADS28 protein has transcriptional activation ability (data not shown), the cDNA sequence encoding GmMADS28∆C lacking the activation domain (185–243) was used to construct the GAL4-BD fusion pDEST32-*GmMADS28∆C* as the bait plasmid and used to screen a yeast cDNA library prepared from soybean flowers. The positive clones were then verified by retransformation and subjected to β-galactosidase assay.

### Availability of supporting data

The sequence of *GmMADS28* has been deposited in GenBank/EMBL under accession number AJ878424. The GenBank/EMBL accession numbers of MADS-box proteins used in the phylogenetic tree are: GRCD1 (AJ400623), GRCD2 (AJ784156), SEP1/AGL2 (M55551), SEP4/AGL3 (P29383), SEP2/AGL4 (M55552), SEP3/AGL9 (AF015552), GmSEP1 (DQ159905), OsMADS1 (L34271), OsMADS5 (U78890), ZMM6 (AJ430692), ZMM27 (AJ430694), VvMADS4 (AF373603), SlSEP3 (BAD10945), LjSEP3 (AY770397).

## Abbreviations

SEP3: *SEPALLATA3*; TFs: Transcription factors; FBP11: *FLORAL BINDING PROTEIN 11*; ORF: Open reading frame; GFP: Green fluorescence protein; DAF: Days after flowering; SEM: Scanning electron microscopy; WT: Wild type; Y2H: Yeast two-hybrid assay; EST: Expressed sequence tag; RACE: Rapid amplification of cDNAs; Ct: Threshold cycle.

## Competing interests

The authors declare that they have no competing interests.

## Authors’ contributions

FH: Conceptualization of experiments, clone and sequence characterization of GmMADS28, functional analysis of GmMADS28, writing of manuscript; GLX: Yeast two-hybrid assay, the expression analysis of genes involved in flowering time or organ identity in transgenic plants; YJC: tobacco transformation, Cell morphological analysis; HCL: Subcellular localization, RNA in situ hybridization; QX: Yeast two-hybrid assay; TJZ: supply of the soybean mutant; JYG: supply of the soybean mutant; DYY: conceptualization of experiments; critical revision of manuscript. All authors read and approved the final manuscript.

## Supplementary Material

Additional file 1: Figure S1 *GmMADS28* expression during seed development. *Actin* gene was used as the reference gene. DAF: days after flowering. Click here for file

Additional file 2: Figure S2 The RT-PCR analysis of 35S:GmMADS28 transgenic plants. P: Positive control, plasmid DNA; WT: wild type plant; 1–13: the 35S:GmMADS28 lines. Click here for file

Additional file 3: Figure S3 The yeast two hybrid assay identified five proteins (GmSIP1 ~ GmSIP5) interacting with GmMADS28. The cDNA sequence coding GmMADS28∆C lacking the activation domain was cloned in pDEST32 and used as a bait to screen cDNA library prepared from soybean flowers. The transformation of pEXP™32/Krev1 and pEXP™22/RalGDS-wt or pEXP™22/RalGDS-m1 served as strong positive and weak positive controls while the transformations of pEXP™32/Krev1 and pEXP™22/RalGDS-m2 or pDEST32 and pDEST22 served as negative controls. Click here for file

Additional file 4: Table S1 The primer sequences used in gene expression analysis. Click here for file
