# Supplementary figures and images for: A soybean MADS-box protein modulates floral organ numbers, petal identity and sterility
Source: BMC Plant Biol. 2014 Apr 2;14:89. doi: 10.1186/1471-2229-14-89 (PMC4021551; doi:10.1186/1471-2229-14-89)

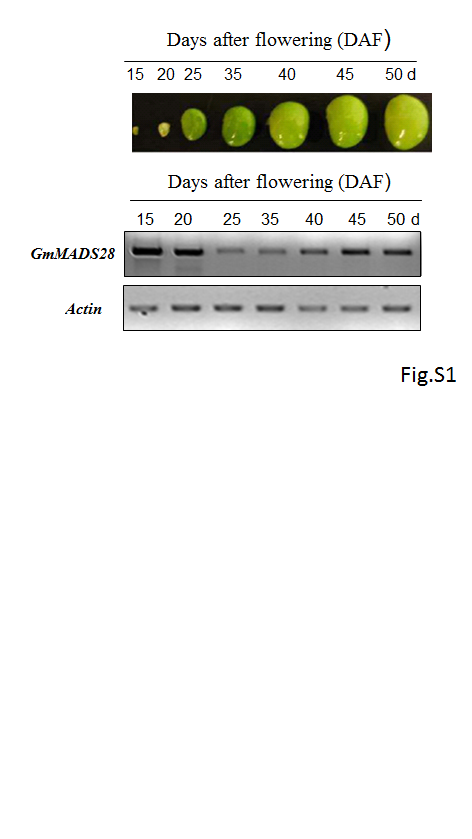

Supplement: Additional file 1: Figure S1 — GmMADS28 expression during seed development. Actin gene was used as the reference gene. DAF: days after flowering. [file 1471-2229-14-89-S1.tiff]

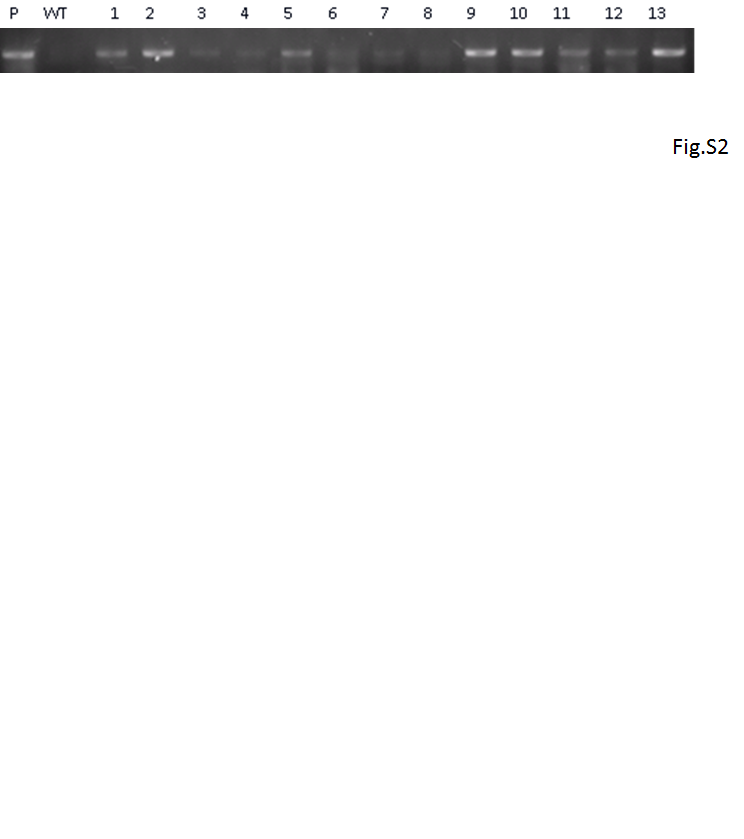

Supplement: Additional file 2: Figure S2 — The RT-PCR analysis of 35S:GmMADS28 transgenic plants. P: Positive control, plasmid DNA; WT: wild type plant; 1–13: the 35S:GmMADS28 lines. [file 1471-2229-14-89-S2.tiff]

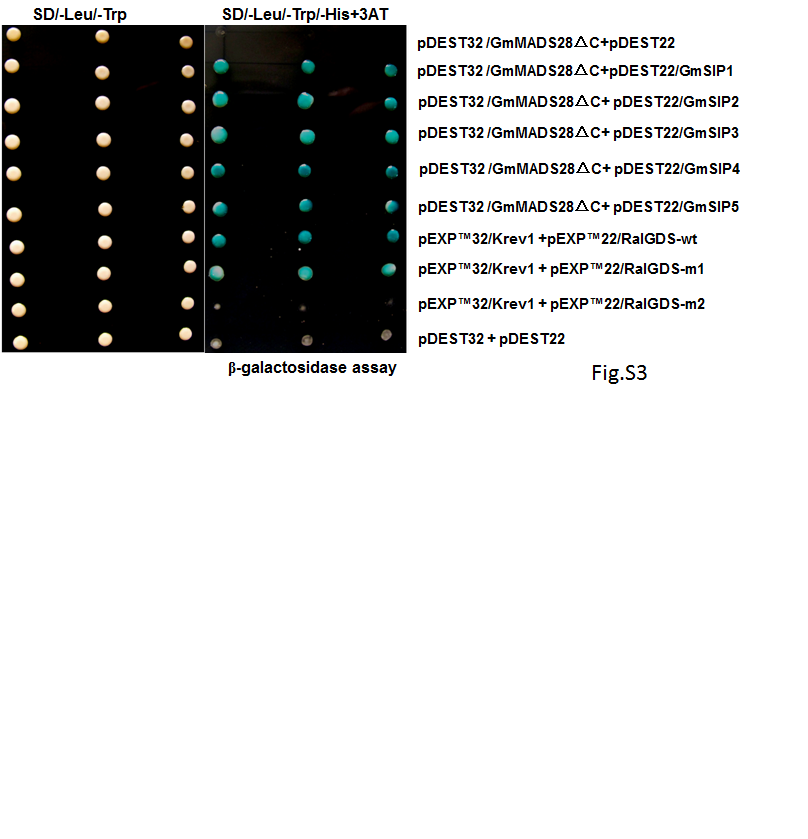

Supplement: Additional file 3: Figure S3 — The yeast two hybrid assay identified five proteins (GmSIP1 ~ GmSIP5) interacting with GmMADS28. The cDNA sequence coding GmMADS28∆C lacking the activation domain was cloned in pDEST32 and used as a bait to screen cDNA library prepared from soybean flowers. The transformation of pEXP™32/Krev1 and pEXP™22/RalGDS-wt or pEXP™22/RalGDS-m1 served as strong positive and weak positive controls while the transformations of pEXP™32/Krev1 and pEXP™22/RalGDS-m2 or pDEST32 and pDEST22 served as negative controls. [file 1471-2229-14-89-S3.tiff]
