# Supplementary material for: A soybean MADS-box protein modulates floral organ numbers, petal identity and sterility
Source: BMC Plant Biol. 2014 Apr 2;14:89. doi: 10.1186/1471-2229-14-89 (PMC4021551; doi:10.1186/1471-2229-14-89)
Supplement: Additional file 4: Table S1 — The primer sequences used in gene expression analysis. [file 1471-2229-14-89-S4.doc]

Table S1 The primer sequences used in gene expression analysis*

| Gene Name | Accession Number | Primer sequences |
| --- | --- | --- |
| *SOC1* | FG643782.1 | 5’-TTCATTTCCTTTGCCTGGTC-3’  5’-CTGAGTTTTTCCCCTCACCA-3’ |
| *LEAFY* | U15798 | 5’-AAGGAGCGAGGAGAGAATGTTG-3’  5’-AGGAGGTAGATATAGGGGCAAGC-3’ |
| *AGL8/FUL* | EB451861 | 5’-CACATTCGCTCAAGCAAGAA-3’  5’-TCTGATATGCTTCCCCAAGG-3’ |
| *AP2* | AJ299252 | 5’-CACCCTACTACCCCATGG AAA T-3’  5’-CTCATCAGAAAACGCACACC-3’ |
| *AP1* | AF009126 | 5'- ATCTTGAGCACCAGCTCGAT-3'  5'- TGTTGCTCCCACTGAGTCTG-3' |
| *PI* | X67959 | 5'- ATGTTGGAAGATGCCCTTGA-3'  5'- CTGAACTCGGAAGGCAAAAG-3' |
| *DEF* | X96428 | 5’-GGGGACTATAACTCTGTGCTTGG-3’  5’-GCTAAAGTCCGACCGATTCAC T-3’ |
| *GLO* | X67959 | 5’-CACAGAGAGCTCATGATGTTGG-3’  5’-CTG AACTCGGAAGGCAAAAG-3’ |
| *AG* | L23925 | 5'- CATGTTGGGTGAATCACTGG -3'  5'- CCCTGGCATCAAGTTCATC-3' |
| *EF-1α* | AF120093 | 5'- TGGACACAGGGATTTCATCA -3'  5'-TGGAATACTTGGGGGTGGTA-3' |

* The genes except *EF-1α* are tobacco homologs of the corresponding Arabidopsis genes.
